# Risk-based screening for the evaluation of atrial fibrillation in general practice (R-BEAT): a randomized cross-over trial

**DOI:** 10.1093/qjmed/hcaf001

**Published:** 2025-01-09

**Authors:** Robert Murphy, Ruairi Waters, Andrew Murphy, Suzanne McDermott, Catriona Reddin, Orlaith Hernon, Naomi Davies, Alberto Alvarez-Iglesias, Eamonn Twomey, Eamon O’Shea, Peter Sloane, Joseph Curran, Aoife Kiely, Caitriona Waters, John Kilraine, Siobhan McDonagh, Adrian Carney, Declan Devane, Martin O’Donnell

**Affiliations:** HRB Clinical Research Facility Galway, School of Medicine, University of Galway, Galway, Ireland; HRB Clinical Research Facility Galway, School of Medicine, University of Galway, Galway, Ireland; Department of General Practice, University of Galway, Galway, Ireland; Turloughmore Medical Centre, Galway, Ireland; HRB Clinical Research Facility Galway, School of Medicine, University of Galway, Galway, Ireland; HRB Clinical Research Facility Galway, School of Medicine, University of Galway, Galway, Ireland; HRB Clinical Research Facility Galway, School of Medicine, University of Galway, Galway, Ireland; HRB Clinical Research Facility Galway, School of Medicine, University of Galway, Galway, Ireland; HRB Clinical Research Facility Galway, School of Medicine, University of Galway, Galway, Ireland; Turloughmore Medical Centre, Galway, Ireland; The Claddagh Medical Centre, Galway, Ireland; Sloane Family Practice, Galway, Ireland; Clonbur Medical Centre, Galway, Ireland; Galway Bay Medical Centre, Galway, Ireland; Seapoint Medical Centre, Galway, Ireland; Main Street Clinic, Galway, Ireland; Prospect Hill Centre, Galway, Ireland; Roscam Medical Practice, Galway, Ireland; School of Nursing and Midwifery, University of Galway, Galway, Ireland; HRB Clinical Research Facility Galway, School of Medicine, University of Galway, Galway, Ireland

## Abstract

**Background:**

The optimal approach to the diagnosis of atrial fibrillation in primary care is unclear.

**Aim:**

To determine if external loop recorder (ELR) screening improves atrial fibrillation detection in community-dwelling adults with a CHA_2_DS_2_-VASc score of greater than two.

**Design:**

Randomized cross-over clinical trial.

**Methods:**

Community-dwelling adults ≥55 years with a CHA_2_DS_2_-VASc score of greater than two, who were deemed suitable for atrial fibrillation screening and oral anticoagulation by their general practitioner were randomly assigned to immediate or delayed ELR monitoring. The intervention period was ELR cardiac monitoring for 1 week and the usual care period was healthcare professional pulse screening and completion of electrocardiogram (ECG) or cardiac rhythm strip if pulse was identified as irregular.

**Results:**

Of the 488 participants randomized, 244 were assigned to the immediate monitoring period (intervention) and 244 were assigned to the delayed monitoring period. Mean (SD) age was 75.0 (7.0) years and 333 participants were women (68%). Atrial fibrillation was detected in 32 of 488 participants (6.6%) in the intervention period versus five of 488 (1%) in the usual care period (absolute difference, 5.53% (3.2–7.9%), *P* < 0.001; number needed to screen 15 (11–23)). Twelve cases (37.5%) of ELR-detected atrial fibrillation were greater than 24 h in duration. Oral anticoagulation was initiated in all participants (*n* = 32).

**Conclusions:**

Among older community-dwelling adults with a CHA_2_DS_2_-VASc score of greater than two, screening with ELR for one week was associated with a 5.5% incremental detection of new atrial fibrillation over usual care.

**Trial registration:**

ClinicalTrials.gov Register: NCT03911986

## Introduction

Undiagnosed atrial fibrillation is a major care-gap in stroke prevention, given that about 25–40% of patients with acute ischaemic stroke and atrial fibrillation are first diagnosed at the time of acute stroke presentation.^1^^,^^2^ In high-risk populations, such as patients with recent unexplained ischaemic stroke, targeted screening for atrial fibrillation is recommended by some guidelines, based on results of randomized clinical trials.^3^^,^^4^ In intermediate-risk populations, however, there is uncertainty about the optimal approach for population selection and choice of screening modality with a number of clinical trials evaluating different approaches to screening intermediate-risk primary prevention populations.^5–10^

Screening for atrial fibrillation may be associated with a reduction in stroke as compared to no screening,^11^ but despite the increasing evidence-base, guideline panels have reported there is insufficient evidence to recommend population-wide screening for atrial fibrillation.^12^^,^^13^

External loop recorder (ELR) monitoring is a non-invasive, renewable, approach to screening with cardiac monitors, and is a diagnostic approach favoured by patients.^14^ Atrial fibrillation detected on an ELR is likely to be of a clinically relevant burden to merit oral anticoagulation.^15^ Randomized clinical trials of ELR screening have shown that there can be difficulties with engaging participants throughout the steps of the screening processes, such as in the mSToPS RCT where approximately a third of patients randomized did not complete the screening process,^5^ and in SCREEN-AF RCT where a quarter of participants who were diagnosed with atrial fibrillation did not begin anticoagulation.^6^ More information is needed to identify better approaches to a simplified screening process that avoids these pitfalls and adequately engages participants in the screening process.

With these considerations in mind, we completed the R-BEAT randomized cross-over controlled trial, which was embedded within general practice and recruited participants whose general practitioner (GP) deemed them suitable for both atrial fibrillation screening and initiation of oral anticoagulation if atrial fibrillation was detected. We sought to determine whether routine use of ELR for one-week increased the detection of atrial fibrillation in an intermediate-risk population (defined by CHA_2_DS_2_-VASc score >2) compared to usual care with healthcare professional pulse screening.

## Materials and methods

We completed a randomized controlled, cross-over, multi-center trial in General Practice comparing ELR monitoring for one week with usual care (pulse screening) in participants aged ≥55 years with CHA_2_DS_2_-VASc >2. The protocol is described in detail on protocols.io (https://www.protocols.io/view/risk-based-community-screening-for-the-evaluation-cmgpu3vn).

Inclusion criteria consisted of participants aged 55 years or older, CHA_2_DS_2_-VASc score of greater than two and attendance at a minimum of one appointment with their GP within the last 12 months. Exclusion criteria included known atrial fibrillation/flutter, pre-specified contraindication to oral anticoagulation, current prescription of oral anticoagulation therapy at treatment doses for the prevention of stroke in atrial fibrillation, unsuitable for anticoagulation therapy or cardiac monitoring in the opinion of the participant’s GP. Participants were randomly assigned using a web-based block randomization with variable block size to the immediate or delayed ELR period assigning individual participants in a 1:1 ratio. Written invitations to participate in the clinical trial were sent out to those identified as potentially eligible by their GP. This invitation contained a description of the research study, educational information on atrial fibrillation and explained the practicalities of the screening procedure. The invitation also explained to participants that their GP had reviewed their medical notes and considered them suitable for the study.

The trial was open-label, with blinded outcome assessment and blinding of statistical analysis. An independent Cardiologist reviewed all AliveCor Kardia device tracings and all abnormal cardiac rhythm strips from ELR.

### Usual care period

In the usual care period of the study, pulse screening was undertaken at baseline assessment and consisted of 2 min pulse measurement by a trained research healthcare professional. In participants who had an irregular pulse identified at baseline pulse check, an ECG or measurement with AliveCor Kardia mobile device was completed, depending on local practice at the General Practice site, to rule-out or diagnose atrial fibrillation. Baseline pulse screening was used to determine primary outcome measure in the usual care group, to avoid contamination of findings from the ELR period in the immediate ELR group.

### Intervention period — external loop recorder

All participants were invited to be fitted with the R-Test ELR device with two electrodes placed on the thorax (Supplementary Figure S1). All participants wore the ELR for a one-week period and returned it to their local General Practice, or, if more convenient for the participant, to the Clinical Research Facility (Galway University Hospital, Galway, Ireland) at the end of the monitoring period.

### Outcome measures

The primary outcome measure was newly detected (and centrally confirmed) cases of atrial fibrillation/flutter >2 min in duration on R-Test, or detected on ECG/Rhythm strip following baseline pulse screen (usual care). Secondary outcome measures included newly detected cases of atrial fibrillation/flutter >2 min in duration resulting in introduction of oral anticoagulant therapy, all new detected cases of atrial fibrillation/flutter of any duration and all new detected cases of cardiac arrhythmia. Clinical outcomes included stroke or major bleeding. We defined sub-optimal adherence to the ELR as use on less than five of seven days. Tolerability of the ELR was defined as the incidence of adverse skin reactions related to the device.

### Statistical analysis

Descriptive statistics were used to describe the baseline characteristics of the study population, the flow of trial participants and the level of missing data. Data were analysed on an intention-to-screen basis. The difference in yield of new atrial fibrillation between intervention and usual care was estimated by calculating the difference in detection rate of new atrial fibrillation during the control period and the intervention period, with each participant acting as their own control, and a formal test for statistical significance was completed using the McNemar test for paired proportions (primary analysis).^16^ Yates’ correction was applied if one cell in the contingency table had a cell count of less than five.^17^ We estimated the odds ratio in the intervention versus control periods, and results were presented as crude rates in each group and odds ratios estimates with associated 95% confidence intervals. We explored whether there was an order-effect, i.e. whether detection differed by early versus delayed ELR periods (P-interaction).

We compared participant characteristics between those with atrial fibrillation versus without atrial fibrillation detected, and by maximum duration of atrial fibrillation episode detected. For the latter analyses, we included all detected durations of atrial fibrillation, including those of durations between 30 s and 2 min, which were not included in the primary outcome measure. Logistic regression analysis was used to determine the association of important covariates with newly detected atrial fibrillation. Pre-specified sensitivity analyses included subgroup analyses by age categories, gender, body mass index category, history of hypertension, prior palpitations, prior completion of pulse check and the range of CHA_2_DS_2_-VASc scores. A *P* values of <0.05 or 95% confidence intervals (CI) that did not include 1.0 was considered statistically significant. A two-sided significance level of 0.05 was used for all analyses. Statistical analysis was performed using R statistical software (Version 4.3.0).

## Results

### Participant characteristics

**Figure 1.**
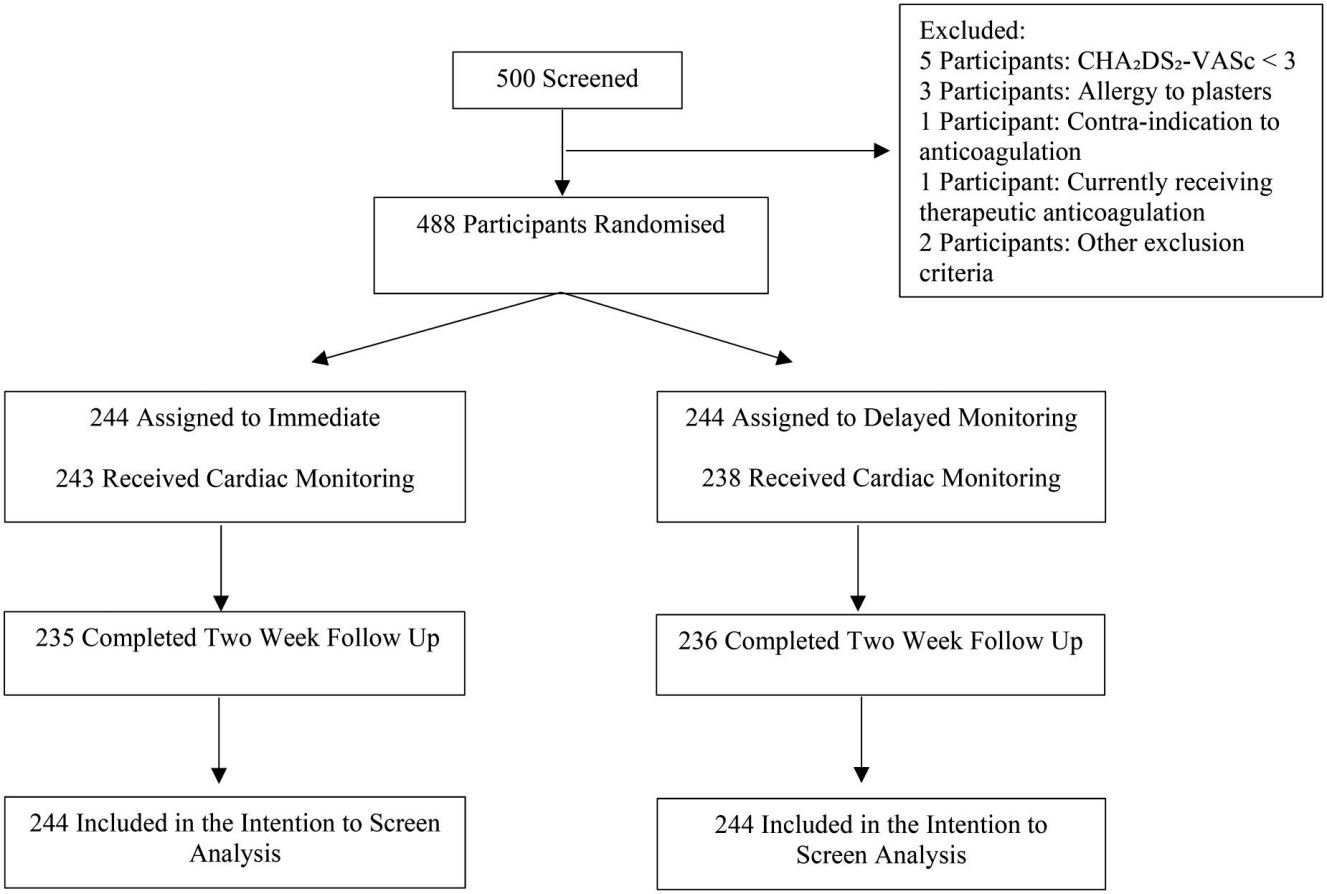
Flow diagram.

From March 2018 to March 2023, 500 participants were recruited, of which 488 participants underwent randomization: 244 were assigned to the immediate monitoring period and 244 were assigned to the delayed monitoring period (Figure 1). Mean (SD) age was 75.0 (7.0) years, 333 participants (68%) were women with a median score of three points (IQR, 3–4 points) on the CHA_2_DS_2_-VASc (congestive heart failure, hypertension, age ≥75 years, diabetes, stroke or transient ischaemic attack, vascular disease, age 65–74 years, sex category) score (Table 1). Age was the most frequent individual components scored in the CHA_2_DS_2_-VASc score (95.1% of participants scoring at least one point for age), followed by a history of hypertension (82.9%) and female gender (68.2%).

**Table 1.** Baseline data by randomization status

Variable	*N*	Overall *N* = 488^a^	Delayed monitoring *N* = 244	Immediate monitoring *N* = 244	*P*-value^b^
Age (mean, SD), years		75.1 (7)	75.0 (6)	75.1 (7)	0.89
Sex	488				
Male		155 (31.8%)	82 (33.6%)	73 (29.9%)	0.38
Female		333 (68.2%)	162 (66.4%)	171 (70.1%)	
Formal education	488				0.017
None		6 (1.2%)	0 (0%)	6 (2.4%)	
Primary		96 (19.7%)	58 (23.8%)	38 (15.6%)	
Second		210 (43.0%)	102 (41.8%)	108 (44.3%)	
Third		148 (30.3%)	73 (29.9%)	75 (30.7%)	
Fourth		28 (5.7%)	11 (4.5%)	17 (7.0%)	
Race	488				
Irish		483 (99.0%)	243 (99.6%)	240 (98.4%)	0.37
Non-Irish		5 (1.0%)	1 (0.4%)	4 (1.6%)	
Baseline measurements					
Systolic BP (mean, SD), mmHg	488	137 (16)	136 (16)	138 (16)	0.36
Diastolic BP (mean, SD), mmHg	488	73 (10)	74 (10)	73 (10)	0.47
Weight (mean, SD), kg	488	77.7 (17)	78.2 (17)	77.1 (17)	0.47
Height (mean, SD), m	488	1.64 (.84)	1.64 (.84)	1.63 (.84)	0.40
BMI (mean, SD)	488	28.9 (5.6)	29.0 (5.7)	28.8 (5.6)	0.78
EQ-5D-5L (median, IQR)	488	80 (70, 90)	80 (70, 90)	80 (70, 90)	0.85
Past medical history					
CHA_2_DS_2_-VASc (median, IQR)	488	3 (3.0-4.0)	3 (3.0-4.0)	3 (3.0-4.0)	0.75
Congestive heart failure	488	13 (2.7%)	6 (2.5%)	7 (2.9%)	0.78
Hypertension	487	405 (83.2%)	211 (86.5%)	194 (79.8%)	0.050
Diabetes	488	115 (23.6%)	59 (24.2%)	56 (22.9%)	0.75
Stroke	488	16 (3.3%)	9 (3.7%)	7 (2.9%)	0.61
TIA	488	37 (7.6%)	19 (7.8%)	18 (7.4%)	0.86
Myocardial infarction	487	24 (4.9%)	14 (5.8%)	10 (4.1%)	0.40
Peripheral arterial disease	487	14 (2.9%)	6 (2.5%)	8 (3.3%)	0.58
Hypercholesterolemia	486	311 (63.9%)	160 (65.8%)	151 (62.1%)	0.47
Hypothyroidism	488	88 (18.0%)	48 (19.7%)	40 (16.4%)	0.35
Hyperthyroidism	488	5 (1.0%)	1 (0.4%)	4 (1.6%)	0.37
Falls/syncope	485	163 (33.6%)	79 (33.0%)	84 (35.0%)	0.61
Obstructive sleep apnoea	488	27 (5.5%)	11 (4.5%)	16 (6.6%)	0.32
Medications					
Number antihypertensive	485				0.52
Zero		84 (17.3%)	37 (15.4%)	47 (19.3%)	
One		159 (32.8%)	81 (33.6%)	78 (31.9%)	
Two or more		242 (49.9%)	123 (51.0%)	119 (48.8%)	
Statin	488	302 (61.9%)	154 (63.1%)	148 (60.7%)	0.58
Anticoagulant	488	3 (0.6%)	0 (0%)	3 (1.2%)	0.25
Antiplatelet	488	196 (39.8%)	102 (41.8%)	94 (38.5%)	0.46
Socioeconomic factors					
Current alcohol consumption	488	340 (69.7%)	162 (66.4%)	178 (73.0%)	0.12
Alcohol intake	355				0.59
Low		270 (76.1%)	131 (78.9%)	139 (73.6%)	
Moderate		55 (15.5%)	23 (13.8%)	32 (16.9%)	
High		30 (8.4%)	12 (7.3%)	18 (9.5%)	
Smoking status	488				0.11
Current		33 (6.8%)	19 (7.8%)	14 (5.7%)	
Former		241 (49.4%)	109 (44.7%)	132 (54.1%)	
Never		214 (43.9%)	116 (47.5%)	98 (40.2%)	
Physical activity	483				0.67
Sedentary		65 (13.5%)	34 (14.0%)	31 (12.9%)	
Mild		202 (41.8%)	103 (42.4%)	99 (41.3%)	
Moderate		204 (42.2%)	102 (42.0%)	102 (42.5%)	
Strenuous		12 (2.5%)	4 (1.6%)	8 (3.3%)	
Employment status	488				0.43
Employed		41 (8.4%)	24 (9.8%)	17 (7.0%)	
Retired/not working		433 (88.7%)	212 (86.9%)	221 (90.5%)	
Other		14 (2.9%)	8 (3.3%)	6 (2.5%)	
Private health insurance	488	310 (63.5%)	155 (63.5%)	155 (63.5%)	>0.99
Private cardiologist	485	89 (18.4%)	46 (18.9%)	43 (17.8%)	0.71
Married	488	281 (57.6%)	148 (60.7%)	133 (54.5%)	

a *n* (%); c(“Mean (SD)”, “Median (IQR)”, “Range”).

b Pearson's Chi-squared test; Wilcoxon rank sum test; Fisher's exact test.

ELR was worn by 481 participants (98.6%), with a median wear time of 7.0 days (IQR, 6.9–7.1 days) and a median analysable time of 6.7 days (IQR, 5.9–7.5 days). Most of the participants (92%) wore the ELR for at least five days, and adherence was similar for the immediate (91%) and delayed groups (92%) (*P* = 0.55). Adverse skin reactions were seen in 27 participants (5.6%) and resolved in all cases.

### Primary outcome measure

**Figure 2.**
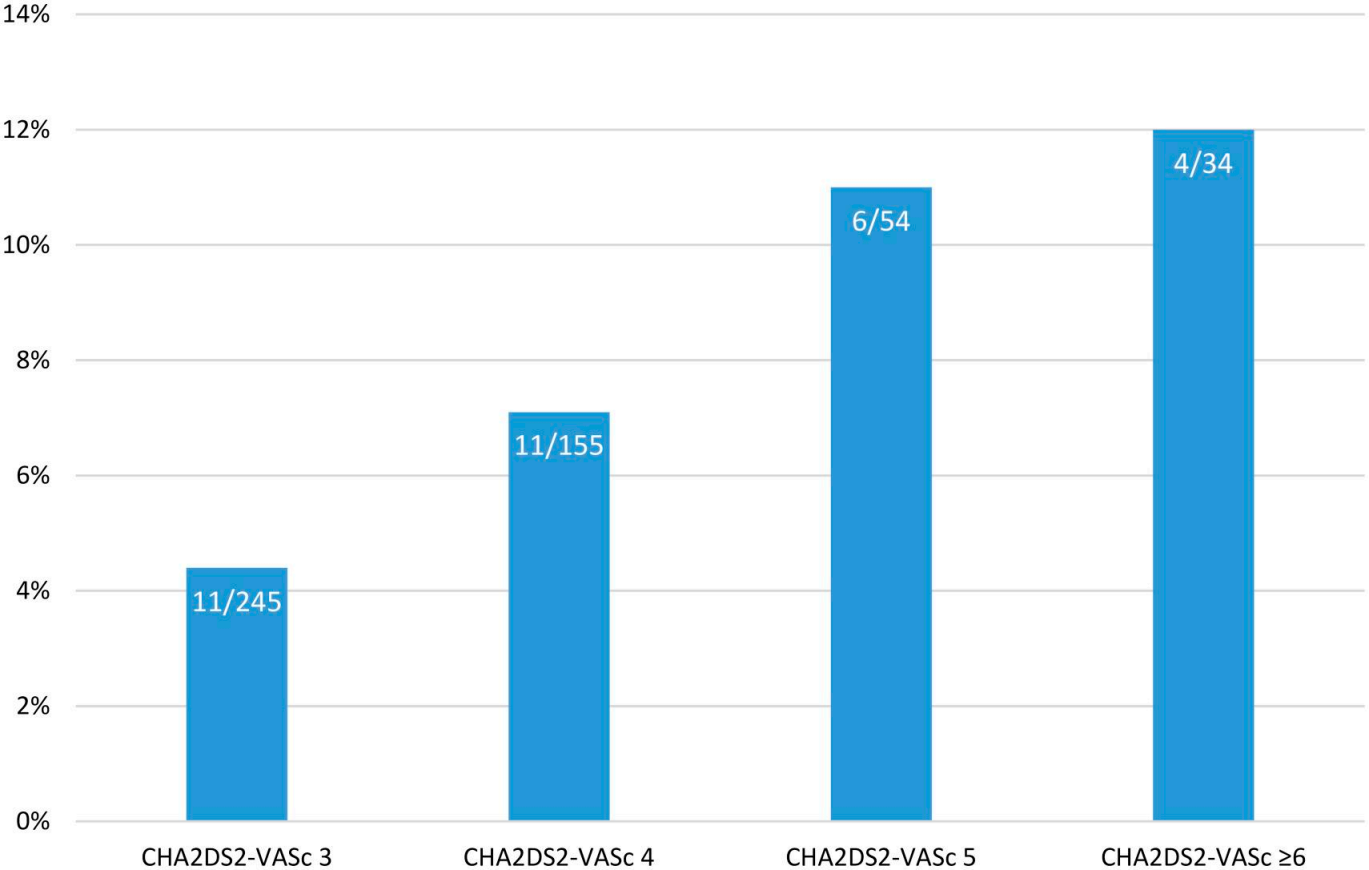
Proportion with atrial fibrillation by CHA_2_DS_2_-VASc score.

In the primary analysis, atrial fibrillation was detected in 32 of 488 participants (6.6%) in the intervention (ELR) period versus five of 488 (1%) in the control period (OR 65 (95% CI 7.02–601.1); *P* < 0.001; absolute difference, 5.53% (2.95–8.10%); *P* < 0.001; number needed to screen 15 (11–23)) (Table 2). There was no significant difference in rates of detected atrial fibrillation between the immediate and delayed monitoring periods (4.5% vs. 8.6%, *P* = 0.06). The incidence of atrial fibrillation rose from 4.5% among participants with a CHA_2_DS_2_-VASc score of three to 11.8% for those with a score of six or more (Figure 2). Patients with atrial fibrillation were older (mean age 79 vs. 75 *P* < 0.001) and had lower scores on the EQ5D5L health related quality of life questionnaire (mean score 71 vs. 76, *P* = 0.03) (Supplementary Table S1).

**Table 2.** Contingency table atrial fibrillation diagnosis by screening strategy

		ELR	
		Atrial fibrillation	No atrial fibrillation
Usual care	Atrial fibrillation	4	1
	No atrial fibrillation	28	455

Among the 32 cases of atrial fibrillation detected with ELR, the duration of the longest episode of atrial fibrillation was between two minutes and 24 h in 20 of these cases and was greater than 24 h in the other 12. Among participants who had atrial fibrillation identified with pulse screening, four of these participants also had atrial fibrillation on ELR, and one participant did not have atrial fibrillation on ELR. This participant without atrial fibrillation on ELR check had been randomized to the delayed monitoring period.

### Secondary outcome measures

All newly detected cases of diagnostic atrial fibrillation resulted in the initiation of oral anticoagulant therapy. There were 137 participants with episodes of atrial fibrillation lasting less than 2 min, considered sub-diagnostic atrial fibrillation (Table 3). Among these sub-diagnostic atrial fibrillation cases, 130 of the 137 cases (94.9%) were less than 30 s in duration.

**Table 3.** Secondary outcomes

Outcome	Proportion
Atrial fibrillation secondary outcomes	
Atrial fibrillation (≥2 min) commencing oral anticoagulation	100% (*n* = 32)
Atrial fibrillation of any duration	34.6% (*n* = 169)
Sub-diagnostic atrial fibrillation (<2 min)	28.1% (*n* = 137)
Non-cardiac rhythm secondary outcomes	
Stroke	0
Major bleeding	0
Adherence—monitor worn for minimum five days	92.1% (*n* = 443)
Tolerability—skin rash	5.6% (*n* = 27)
Cardiac rhythm secondary outcomes	
Multiple premature atrial contractions (PAC)	12.2% (*n* = 59)
Supra-ventricular tachycardia (SVT)	45.9% (*n* = 221)
Ventricular tachycardia (VT)	4.2% (*n* = 20)
Pause less than six seconds	8.7% (*n* = 42)
Other arrhythmia apart from multiple PAC, SVT, VT or pause	36.6% (*n* = 176)
Any arrhythmia R-Test—excluding atrial fibrillation	65.9% (*n* = 317)

**Figure 3.**
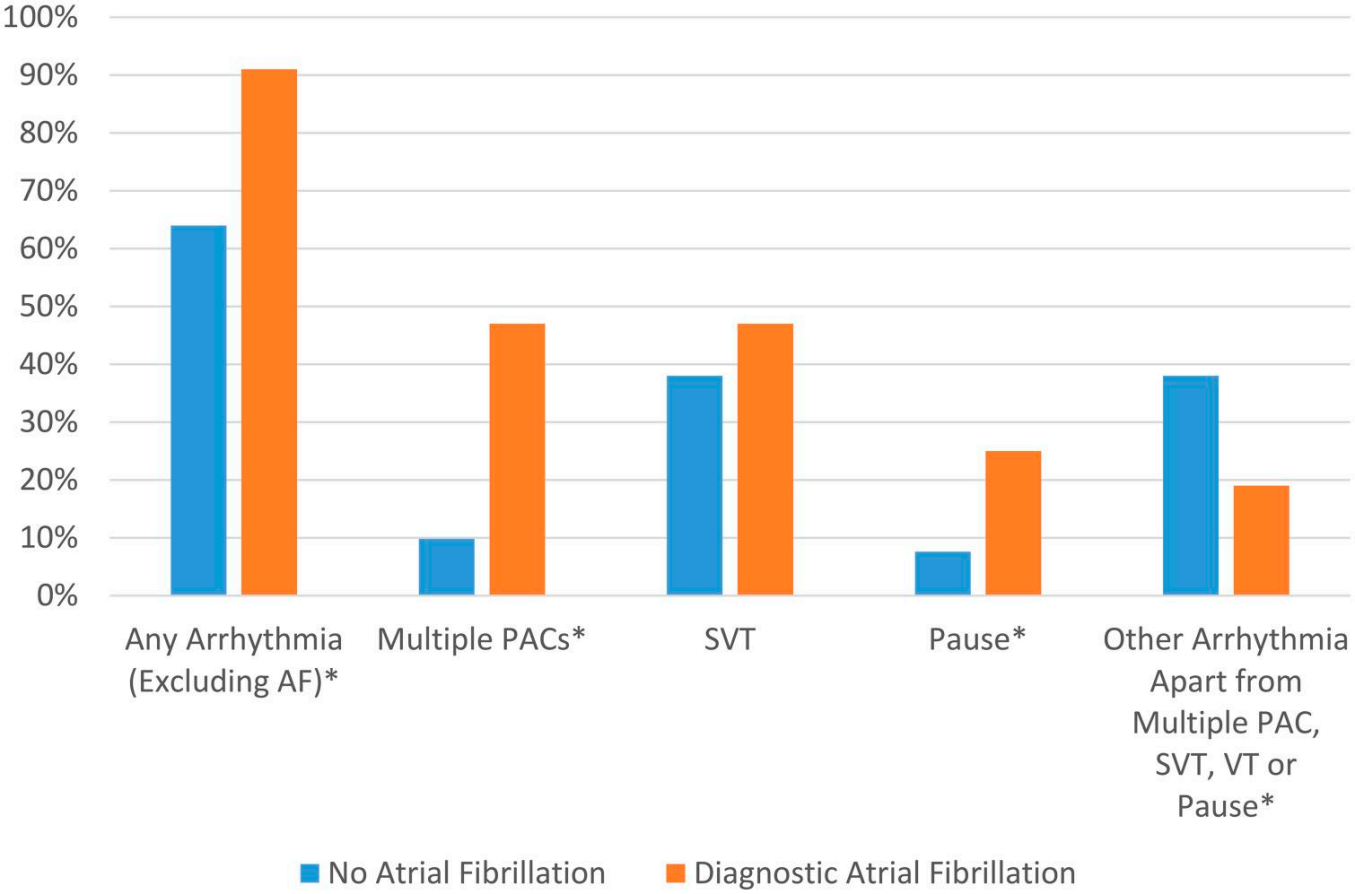
Cardiac arrhythmias according to atrial fibrillation status. *AF = atrial fibrillation; PAC = premature atrial contractions, SVT = supra-ventricular tachycardia, VT = ventricular tachycardia.

Other cardiac rhythm abnormalities on ELR were reported in 66% of participants, including episodes of supra-ventricular tachycardia in 46%, multiple PACs in 12% and VT in 4.2%. Pauses were identified in 8.7% of participants (*n* = 42), but none were symptomatic or greater than 6 s in duration. Participants with atrial fibrillation greater than 2 min in duration were more likely to also be diagnosed with another abnormality on ELR with multiple PACs (47%) and cardiac pauses (25%) being the most frequently encountered (Figure 3).

No stroke or major bleeding events occurred during the two-week trial period. There were no participants who experienced major bleeding during the two-week trial period.

Increasing age and higher CHA_2_DS_2_-VASc scores were associated with a higher odds of atrial fibrillation (OR 1.12 (95% CI 1.05–1.19) per year increased in age; OR 1.47 (1.03–2.08) per unit increase in CHA_2_DS_2_-VASc score). There was no difference in the odds of atrial fibrillation by gender, or prior history of hypertension, palpitations or prior completion of pulse check (Supplementary Table S2).

## Discussion

The use of an ELR for one week among community-dwelling adults at intermediate risk of atrial fibrillation (based on CHA_2_DS_2_-VASc score >2) was associated with an increased detection of atrial fibrillation, compared to usual care which consisted of baseline pulse check with cardiac rhythm assessment if pulse check was irregular. Overall, atrial fibrillation (≥2-min duration) was detected in 6.6% of participants in the ELR period compared with 1% in the control period. This corresponded to a number needed to screen of 15 participants with ELR for one new diagnosis of atrial fibrillation. There was high adherence to the ELR with a median wear time of seven days, with just 5.6% developing an adverse skin rash, which all resolved. All participants with a new diagnosis of atrial fibrillation (≥2 min) were commenced on anticoagulation.

The R-BEAT trial adds to the evidence informing approaches to screening intermediate-risk population. The setting of our trial differed from prior randomized controlled trials evaluating wearable cardiac monitors, as we embedded participant selection within general practice, with clinical oversight by their treating GP, to ensure the detection of atrial fibrillation would likely result in a change in management.^5^^,^^6^^,^^8^^,^^9^^,^^18–20^ Our choice of intervention, a reusable ELR, has implications for the sustainability of the screening approach in contrast to other single use ELRs (e.g. Zios patch), and with the right investment has the potential to be scaled up to be used in a similar fashion to successful improvements in the use of 24-h ambulatory blood pressure monitors (ABPM) within General Practice.^21^ Examining the yield from a similar scale-up of reusable ELR infrastructure in General Practice would be important for the implementation of a reproducible atrial fibrillation screening pathway.

Most atrial fibrillation screening studies that have focused on individual-level recruitment (direct-to-participants), rather than through General Practice, have found that those who participate in screening tend to be younger, of a higher socioeconomic status, and have fewer comorbidities than participants who decline screening, meaning that such populations may not be representative of a general adult population.^5^^,^^22^^,^^23^ Our decentralized approach in R-BEAT aimed to minimize the potential for this selection bias. Pre-selection of suitable participants by their GP in R-BEAT resulted in over 98% of randomized participants completing their cardiac monitoring, significantly higher than that in direct-to-participant atrial fibrillation screening studies where the rate of completion of cardiac monitoring after randomization has ranged from 65% to 84%.^5^^,^^24^

A key finding from R-BEAT was that all participants with diagnostic atrial fibrillation were commenced on oral anticoagulation. Other screening studies, which were not embedded within general practice, such as in pharmacy settings, have not had the same high rate of anticoagulation.^5^^,^^25^ High rates of anticoagulation may also reflect the use of the CHA_2_DS_2_-VASc score, where we selected a threshold of greater than two, which meant that participants diagnosed with atrial fibrillation were automatically eligible for oral anticoagulation.

A high proportion of participants in R-BEAT had additional abnormalities detected on ELR monitoring. Just over a quarter of the participants recruited had evidence of sub-diagnostic atrial fibrillation (defined as a burden less than 2 min), which is associated with a low stroke risk that does not warrant anticoagulation,^26^^,^^27^ but may warrant repeat monitoring in the future to assess for progression to clinical atrial fibrillation. Uncertainty exists at present about how best to structure such repeat monitoring, however as ELRs are reusable they represent an attractive option for interval cardiac monitoring.

Strengths of our trial include the use of reusable medical devices embedded within general practice care, an approach that is expected to include a more diverse population, including those who may be unsuited to the use of a smartphone or direct-to-participant approaches where a participant has to self-apply a monitor (e.g. those with cognitive impairment or lower income). We used a cross-over design to increase the proportion of participants undergoing ELR screening, which increases event rates. Each participant served as their own control and this design reduces the potential for baseline imbalances that may result in confounding. An active comparator where all participants receive the intervention while maintaining the randomization comparison, reduces the risk of contamination, which may occur if participants were randomized to no ELR, and then sought this out in clinical practice. A limitation of this study is that the majority of patients were Caucasian which may limit generalizability. Additionally, our trial was underpowered to detect differences in outcomes such as stroke or bleeding events.

## Conclusions

In conclusion, the R-BEAT RCT demonstrated that among older, community-dwelling adults attending General Practice with a CHA_2_DS_2_-VASc score of greater than 2, screening with ELR for one week was associated with a 5.5% increase in the detection of new atrial fibrillation, compared to usual care. The screening was well tolerated and resulted in the initiation of anticoagulation therapy in all newly detected cases.

## Supplementary Material

hcaf001_Supplementary_Data

## Data Availability

Data from this study may be requested from the corresponding author upon reasonable request. De-identified participant data and the data dictionary can be requested. Specific requests for data will require the submission of a proposal with a specific research question and will require a data access agreement to be signed.

## Transparency

The lead authors affirm that the manuscript is an honest, accurate, and transparent account of the study being reported and that no important aspects of the study have been omitted.
