# Supplementary figures and images for: Risk-based screening for the evaluation of atrial fibrillation in general practice (R-BEAT): a randomized cross-over trial
Source: QJM. 2025 Jan 9;118(3):166–73. doi: 10.1093/qjmed/hcaf001 (PMC12051387; doi:10.1093/qjmed/hcaf001)

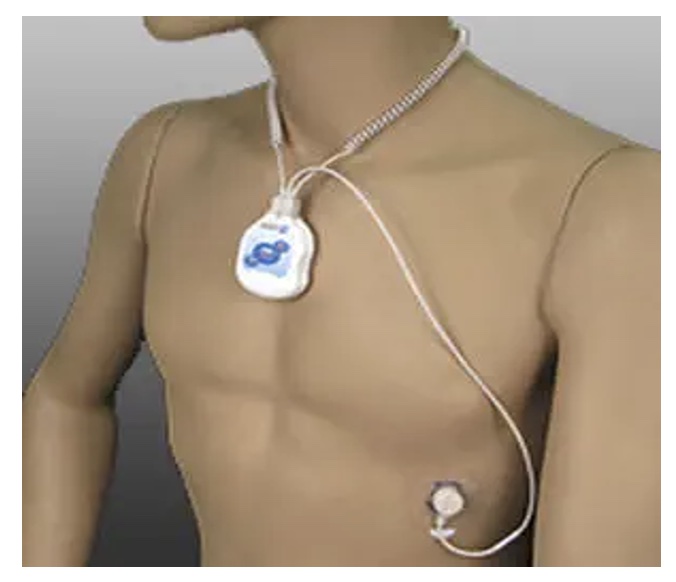

Supplement: hcaf001_Supplementary_Data [file hcaf001_supplementary_data.zip › hcaf001_Supplementary_Data/Supplementary_Figure1.jpg]
